# Structural and functional modelling of SARS-CoV-2 entry in animal models

**DOI:** 10.1038/s41598-020-72528-z

**Published:** 2020-09-28

**Authors:** Greg N. Brooke, Filippo Prischi

**Affiliations:** School of Life Sciences, University of Essex, Colchester, CO4 3SQ UK

**Keywords:** Molecular modelling, Viral proteins, Viral infection

## Abstract

SARS-CoV-2 is the novel coronavirus responsible for the outbreak of COVID-19, a disease that has spread to over 100 countries and, as of the 26th July 2020, has infected over 16 million people. Despite the urgent need to find effective therapeutics, research on SARS-CoV-2 has been affected by a lack of suitable animal models. To facilitate the development of medical approaches and novel treatments, we compared the ACE2 receptor, and TMPRSS2 and Furin proteases usage of the SARS-CoV-2 Spike glycoprotein in human and in a panel of animal models, i.e. guinea pig, dog, cat, rat, rabbit, ferret, mouse, hamster and macaque. Here we showed that ACE2, but not TMPRSS2 or Furin, has a higher level of sequence variability in the Spike protein interaction surface, which greatly influences Spike protein binding mode. Using molecular docking simulations we compared the SARS-CoV and SARS-CoV-2 Spike proteins in complex with the ACE2 receptor and showed that the SARS-CoV-2 Spike glycoprotein is compatible to bind the human ACE2 with high specificity. In contrast, TMPRSS2 and Furin are sufficiently similar in the considered hosts not to drive susceptibility differences. Computational analysis of binding modes and protein contacts indicates that macaque, ferrets and hamster are the most suitable models for the study of inhibitory antibodies and small molecules targeting the SARS-CoV-2 Spike protein interaction with ACE2. Since TMPRSS2 and Furin are similar across species, our data also suggest that transgenic animal models expressing human ACE2, such as the hACE2 transgenic mouse, are also likely to be useful models for studies investigating viral entry.

## Introduction

Coronaviruses are a large family of viruses that can cause respiratory diseases in humans. These can be mild, for example the common cold, but some coronaviruses have caused severe respiratory disease outbreaks in recent years. This family of viruses were found to be the cause of the 2002 Severe Acute Respiratory Syndrome (SARS coronavirus, SARS-CoV) and 2012 Middle East Respiratory Syndrome (MERS coronavirus, MERS-CoV) outbreaks^1^. In December 2019, a novel coronavirus (SARS-CoV-2) was identified in Wuhan City of Hubei Province (China) in patients that had developed viral pneumonia, called COVID-19^2^.

SARS-CoV-2 is a positive-sense single-stranded RNA virus, that belongs to the *β-coronaviruses* family along with SARS and MERS^3^. Sequencing of the genome of SARS-CoV-2 has demonstrated that it is closely related to coronaviruses isolated from bats. Indeed, these analyses have shown that the genome of SARS-CoV-2 has 96.1% sequence similarity with SARSr-Ra-BatCoV-RaTG13, identified in *Rhinolophus affinis* bats captured in Pu’er (China) in 2013^2,4^. For this reason, it has been proposed that bats have acted as an ecological reservoir for SARS-CoV-2. However, since humans have limited contact with bats, it is believed that SARS-CoV-2 passed through an intermediate host before spilling over into the human population. It remains unclear as to which species may have acted as the intermediate host. In the case of SARS, bats were also likely to have been the ecological reservoir and farmed Civet cats were suggested to have been the intermediate species, although several studies have disputed this^5^.

The SARS-CoV-2 genome contains five genes that code for four structural proteins—*spike* (S), *envelope* (E), *membrane* (M) and *nucleocapsid* (N)—and 16 non-structural proteins^6^. Viral entry into human cells is mediated by an interaction between the S glycoprotein and the Angiotensin-Converting Enzyme 2 (ACE2) receptor^7^. ACE2 is a metalloprotease that lowers blood pressure by catalysing the hydrolyses of angiotensin II^8^. However, ACE2 enzymatic activity is not related, or needed, in SARS-CoV-2 entry into the host cells. Importantly, cells lacking ACE2 are not susceptible to SARS-CoV-2 infection^9^. Cryo–electron microscopy analysis of human ACE2 bound to the Receptor Binding Domain (RBD) of the SARS-CoV-2 S glycoprotein showed that ACE2 is a dimer that interacts with two S protein trimers^10^. Detailed structural data have also shown that the S protein binds human ACE2 with high affinity (~ 15 nM). This is 10–20 times higher than the affinity between the SARS-Cov S protein and ACE2, which likely explains the high infectivity of SARS-CoV-2^11^.

Upon binding of the S protein to ACE2, the S protein goes through a conformational change that exposes a cleavage site between the S1 and the S2 domains, which is cleaved initially by Furin and then by the Transmembrane Serine Protease 2 (TMPRSS2)^12–14^. S2 is then further cleaved at the S2′ position which exposes the fusion peptide, promoting endocytic entry of the virus^15^. Furin is an ubiquitously expressed type I transmembrane serine-protease, which has been intensely investigated for its roles in activation of substrates, bacterial and viral infections, as well as cancer and metastasis^16,17^. The extracellular region of Furin contains a subtilisin-like catalytic domain and a calcium-dependent regulatory P domain^18^. The subtilisin-like domain contains the histidine, aspartate, and serine residues (the catalytic triad) necessary for enzymatic activity^18^.

TMPRSS2 is a type II transmembrane serine proteases (TTSPs) which has been found to co-express, co-localise and interact with ACE2^14^. TMPRSS2 belongs to the trypsin (S1) fold subfamily, which is characterised by a highly conserved catalytic serine protease domain stabilised by three intradomain disulphide bonds. Similarly to the Furin subtilisin-like domain, the peptidase S1 domain contains the catalytic triad necessary for enzymatic activity^19^. Interestingly, studies on SARS-CoV have shown that the binding of the S protein to ACE2 also induces cleavage of ACE2 by TMPRSS2, and it has been suggested that the SARS-S-induced shedding of ACE2 may increase uptake of viral particles^20^.

One of the major challenges for the study of SARS-CoV-2, and for the development of effective COVID-19 vaccines and treatments, is the lack of appropriate animal models. Multiple animals have been shown to be experimentally susceptible to SARS-CoV (e.g. macaques, cats, ferrets, guinea pigs and civet cats)^21–25^. Similarly, SARS-CoV-2 has been shown to infect multiple animal species and a number of model systems have been proposed for the study of COVID-19^26^. For example, Shi et al.^27^ found that SARS-CoV-2 can replicate in dogs, pigs, chicken and ducks, although viral replication in these animals is relatively weak. In contrast, the same group found that the virus can replicate efficiently in ferrets and cats.

It remains to be fully elucidated as to why infection rates differ between species, but structural differences in the viral entry receptors are likely to be important. Here we have used a combination of bioinformatics approaches to compare the binding of the SARS-CoV and SARS-CoV-2 S proteins to ACE2 and the structures of TMPRSS2 and Furin in a selected group of animal models; namely mouse, rat, guinea pig, rabbit, ferret, cat, dog hamster and macaque. Our results suggest that macaque, ferret and hamster represent the most promising animal models for the study of ACE2 inhibitors.

## Methods

### ACE2 structures preparation and docking

The 3D structures of the SARS-CoV-2 and SARS-CoV RBD, in complex with human ACE2 (hACE2), were retrieved from the RCSB Protein Data Bank (PDB ID 6M17 and 2AJF respectively^10,28^). FASTA sequences were retrieved from NCBI (Table 1). ACE2 homology models were generated using Swiss Model^29^ and the hACE2 structure as a template. GROMACS 2019.3^30^ with AMBER99SB-ILDN force field was used to resolve high energy intramolecular interaction and remove modelling biases before docking simulations. Structures were centred in a cubic box filled with TIP3P water molecules and counter ions. Simulations were run applying periodic boundary conditions. The energy of the system was minimised with 10,000 steps using a steepest descent algorithm and equilibrated by running 100 ps of NVT (using V-rescale temperature coupling with tau-t of 0.1) and 100 ps NPT (applying Berendsen pressure coupling setting a tau-p of 0.5). ACE2 docking simulations with the RBD of the SARS-CoV-2 S glycoprotein were performed using the web server version of HADDOCK^31^ (https://haddock.science.uu.nl). The docking simulations were driven using the binding interface derived from the RBD-ACE2 structures (PDB ID 6M17 and 2AJF) using PDBePISA^32^. The structures of the RBD-ACE2 complexes are available on https://github.com/fprischi/Supplementary_ComplexesStructures. ACE2-RBD complexes structures were compared using PDBePISA, LigPlot + v2.2 and PyMol^33–35^. ACE2 N-glycosylation sites were retrieved from UniProt^36^. The electrostatic surface potential was calculated and visualised using the PyMol Adaptive Poisson-Boltzmann Solver (APBS) package (https://pymolwiki.org/index.php/APBS). All structures were visualised, and relative figures prepared, using PyMol^35^ (The PyMOL Molecular Graphics System; https://www.pymol.org).

**Table 1:** Summary of species included in the study.

Species	Name	ACE2		TMPRSS2		Furin	
*Homo sapiens*	Human	hACE2	NP_001358344.1	hTMPRSS2	NP_005647.3	hFurin	NP_002560.1
*Cavia porcellus*	Guinea pig	cavACE2	XP_023417808.1	cavTMPRSS2	XP_013001527.1	cavFurin	XP_013014060.1
*Canis lupus familiaris*	Dog	dogACE2	NP_001158732.1	dogTMPRSS2	XP_022268981.1	dogFurin	XP_022272656.1
*Felis catus*	Cat	catACE2	XP_023104564.1	catTMPRSS2	XP_023094477.1	catFurin	XP_023110662.1
*Rattus norvegicus*	Rat	ratACE2	NP_001012006.1	ratTMPRSS2	NP_569108.2	ratFurin	XP_008757777.1
*Oryctolagus cuniculus*	Rabbit	rabACE2	XP_002719891.1	rabTMPRSS2	XP_008250697.1	rabFurin	XP_002721548.2
*Mustela putorius furo*	Ferret	ferACE2	NP_001297119.1	ferTMPRSS2	XP_012916721.1	ferFurin	XP_004763757.1
*Mus musculus*	Mouse	musACE2	NP_081562.2	musTMPRSS2	NP_056590.2	musFurin	NP_035176.1
*Cricetulus griseus*	Hamster	hamACE2	XP_003503283.1	hamTMPRSS2	XP_027271516.1	hamFurin	NP_001230915.1
*Macaca fascicularis*	Macaque	macACE2	XP_005593094.1	macTMPRSS2	XP_005548700.1	macFurin	XP_005595531.1

Species names and abbreviation list with corresponding NCBI accession codes for ACE2, TMPRSS2 and Furin.

### TMPRSS2 structures preparation

TMPRSS2 FASTA sequences were retrieved from NCBI (Table 1). The human TMPRSS2 (hTMPRSS2) model was generated using I-TASSER^37^. The structures of TMPRSS2 are available on https://github.com/fprischi/Supplementary_TMPRSS2. For consistency with the ACE2 models, the human structure that we modelled here was used as a template for homology modelling of TMPRSS2 for the other species using Swiss Model^29^. Structures were compared, analysed and visualised using PyMol^35^ (The PyMOL Molecular Graphics System; https://www.pymol.org).

### Furin sequence analysis

Furin FASTA sequences were retrieved from NCBI (Table 1) and aligned using Clustal Omega^38^.

## Results

### ACE2 interaction with the SARS-Cov-2 spike protein differs between species

**Figure 1:**
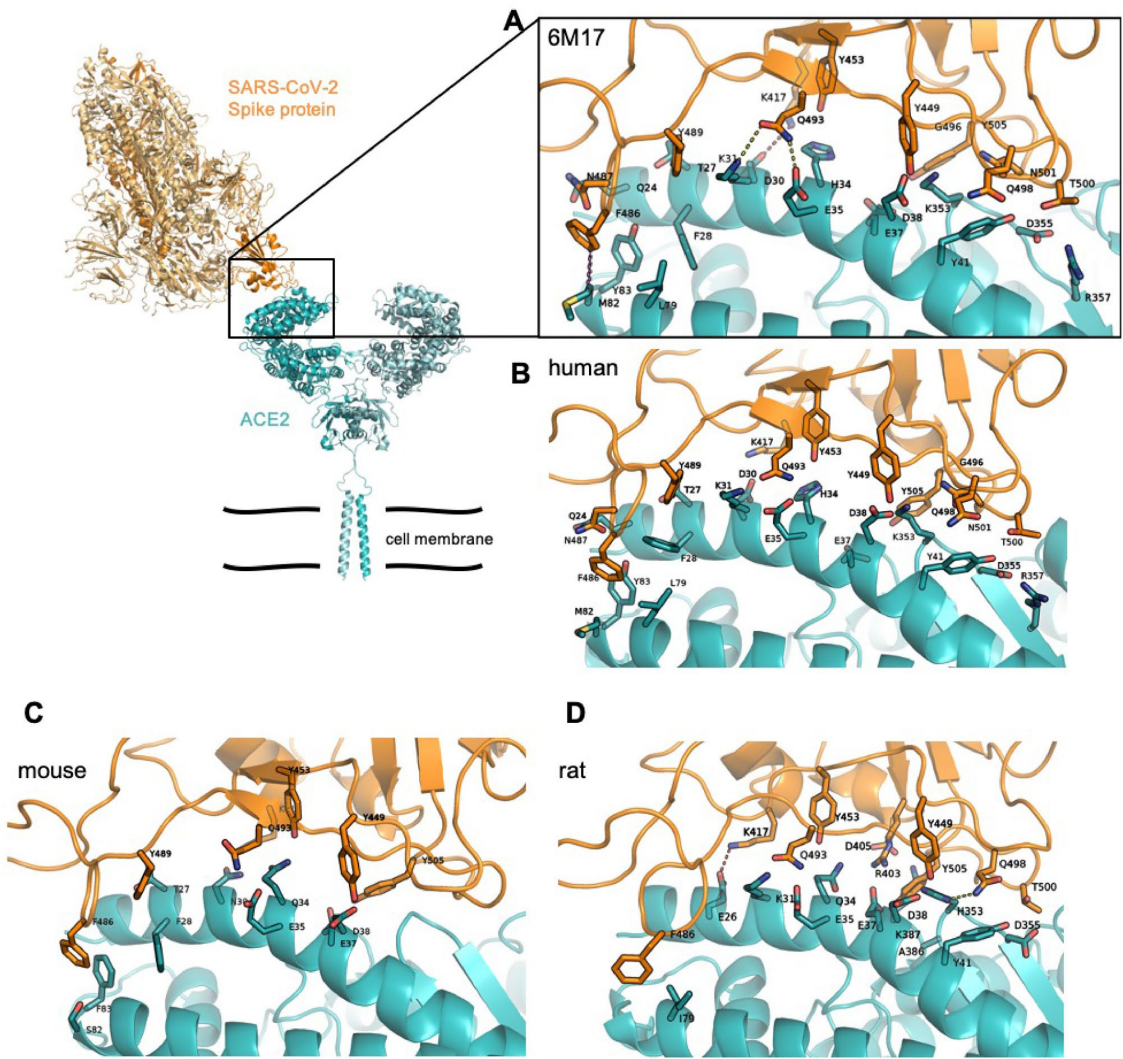
ACE2 PD – SARS-CoV-2 S protein RBD interaction surface. Cartoon representation of the trimeric SARS-CoV-2 S protein (PDB ID 6VSB) with the human ACE2 dimer (PDB ID 6M17), with the S protein in orange and the ACE2 in teal. In the close-up panels, the residues involved in direct interactions (see Table 2) are shown as sticks, with the SARS-CoV-2 RBD in orange and the ACE2 in teal (cryo-EM structure (**A**), human model (**B**), mouse model (**C**)**,** rat model (**D**)). H-bonds, salt bridges and hydrophobic interactions are shown as yellow, red and purple dotted lines respectively. The Q498-H353 H-bond and K417-E26 slat bridge present only in the rat complex are shown with a yellow and red dotted line respectively. All structures are in the same orientation.

ACE2 is a zinc carboxypeptidase type I transmembrane protein, with an extracellular N-terminal peptidase domain (PD) and a cytosolic C-terminal collectrin-like domain (CLD) (Figure S1). The receptor binding domain (RBD) of the SARS-CoV-2 S protein binds directly to the ACE2 PD, and analysis of the crystal structure of this complex shows that the interaction is mostly driven by polar interactions (Table 2). Of particular interest are two key hydrogen bonds between ACE2 K31/E35 and S protein RBD Q493, the salt bridge between ACE2 D30 and RBD K417, and the hydrophobic interaction between ACE2 M82 and RBD F486 (Fig. 1A)^28,39–41^. Interestingly, Q493, K417 and F486 are not conserved between SARS-CoV and SARS-CoV-2, and these differences are linked to the higher affinity of the SARS-CoV-2 S protein for ACE2^12^.

**Table 2:** Residues forming direct interactions in the ACE2 PD – SARS-CoV-2 S protein RBD complexes.

	ACE2—RBD (PDB 6M17)			hACE-RBD	cavACE2	dogACE2	catACE2	ratACE2	rabACE2	ferACE2	musACE2	hamACE2	macACE2
	α1 helix	α2 helix	β3-β4 loop										
H-bond/polar interactions	Q24-N487, T27-Y489, **K31-Q493**, H34-Y453, **E35**-**Q493**, E37-Y505, D38-Y449, Y41-T500, Y41-N501, Y41-Q498		K353-G496, D355-T500, R357-T500	Q24-N487, T27-Y489, K31-Q493, H34-Y453, E35-Q493, E37-Y505, D38-Y449, Y41-T500, Y41-N501, Y41-Q498, K353-G496, D355-T500, R357-T500	Q24-N487, T27-Y489, E31-Q493, K34-Y453, D38-Y449, Y41-Q498, G352-Q498, K353-Y449, K353-Y495, K353-G496, K353-Q498, N354-T500, D355-T500, A386-Y505	T27-F456, K31-Q493, Y34-Y453, E35-S494, Y41-T500, Y41-N501, Q42-Y449, Y83-N487, Y83-Y489, E326-V503, N330-T500, K353-Y505, D355-T500, R357-T500	T27-Y473, T27-Y489, K31-Q493, H34-Y453, E35-Q493, E37-Y505, E38-Y449, Y41-Q498, Y83-N487, K355-N501, K355-G496, R395-Y505	K31-Q493, Q34-Y453, E35-Q493, D38-Y449, Y41-Q498, H353-Q498, D355-T500, A386-Y505	T27-Y489, K31-Q493, E35-Q493, D38-Y449, E37-Y505, Y41-Q498, Y41-T500,	T27-Y489, F28-Y489, K31-Q493, E35-Q493, E37-Y505, E38-Y449, Y41-T500, K353-Y495	T27-Y489, N30-K417, Q34-Y453, E35-Q493, E37-Y505, D38-Y449	Q24-N487, K31-Q493, Q34-Y453, E35-Q493, D38-Y449, K353-Y495, A386-Y505	Q24-G476, K31-Q493, E35-S494, E35-Q493, E37-Y505, D38-Y449, Y41-T500, Y83-N487, K353-G496
Salt bridge	**D30-K417**			D30-K417	D30-K417, K35-E484	E30-K417	E30-K417	E26-K417, E37-R403, K387-D405	E30-K417	E30-K417		E30-K417	E30-K417
Hydrophobic	**F28-F486**, F28-Y489	**L79-F486, M82-F486, Y83-F486**		F28-F486, F28-Y489, L79-F486, M82-F486, Y83-F486	L79-F486	L24-A475, L24-G476, Y83-F486	L24-A475, L24-G476, T82-F486	I79-F486	L24-A475, L24-G476, Y83-F486	F28-F486, L24-G476, L24-A475, L24-F456, Y34-Y453, Y34-L455, H79-F486, F83-F486	F28-Y489, S82-F486, F83-F486	L79-F486, N82-F486	T27-F456, F28-Y489, L79-F486, M82-F486
	Suitable model system				✕	✓	✓	✕	✕	✓	✕	✓	✓

Residues forming contacts in the EM structure (PDB ID: 6M17) and in the HADDOCK docking models are listed by their position and by their single-letter identity, with the first residue belonging to ACE2 and the second to the S protein. Interactions were identified using PyMol and PDBePISA.

In order to predict if the SARS-CoV-2 S protein binds ACE2 of other animal species, focusing mostly on laboratory model systems, we generated homology models for cavACE2, dogACE2, catACE2, ratACE2, rabACE2, ferACE2, musACE2, hamACE2 and macACE2 (Table1). Alignment of the ACE2 sequences from these species, revealed a high conservation, with a sequence identity between 77.2% and 95.6% (Supplemental Figure S1-2). This allowed us to produce reliable ACE2 PD models via homology modelling using the hACE2 as a template. Importantly, before performing docking simulations, homology modelling biases were removed via molecular dynamics equilibration. We then ran restrained docking simulations between the ACE2 PD models and the SARS-CoV-2 S protein RBD to generate optimised complexes. A docking simulation using hACE2 as a control was also performed (Fig. 1B). The presence of a similar network of interactions in the docking output for the hACE2 simulation compared to the one observed in the EM structure was used to validate the approach adopted (Table 2). Further validation of our approach comes from the RBD-macACE2 complex (Supplementary Figure S3). The level of sequence identity between hACE and macACE2 is 95.6% (Supplemental Figure S2) and our modelling show that the RBD-hACE2 and RBD macACE2 complexes are overall very similar, in line with experimental data showing that SARS-CoV-2 replication and shedding and disease symptoms are similar in human and macaque^42,43^.

Overall the hydrophobic contributions that stabilise the RBD-ACE2 complex are similar in all models, with the ferACE2-RBD having a slightly higher number of hydrophobic contacts (Table 2 and Supplementary Figure S3). Interestingly, M82 in the hACE2 is not conserved across species (Supplemental Figure S1), and only the hACE2, hamACE2, macACE2, catACE2 and musACE2 form hydrophobic interaction between residue 82 and the RBD F486 (Supplemental Figure S3). However, RBD F486 is in contact, in all complex structures, with a relatively hydrophobic patch formed by the ACE2 residues 28, 79 and 83 (Supplemental Figure S4A). Comparison of the surface electrostatic potentials of the ACE2 models identified a similar distribution of charges on the α1 helix, α2 helix and β3-β4 loop across all species (Supplementary Figure S5).

Differences between the structure of hACE2 and musACE2 have been previously described to explain why SARS-CoV is a mild infection in mice^44^. The most strikingly difference between the hACE2 and musACE2 are the D30 to N30 and K31 to N31 substitutions. This results in the lack of a salt bridge and a key H-bond in the musACE2-SARS-CoV-2 RBD complex (Table 2 and Fig. 1C). Specifically, the salt bridge with the K417 of the RBD seems to be a major driver of the interaction. In fact, similar to musACE2, ratACE2 has an Asn in position 30, which prevents formation of a salt bridge with K417 in the RBD. However, E26 in the ratACE2 forms a salt bridge with K417, resulting in an altered complex structure with a shift of 6.5 Å of the RBD over the ratACE2, compared to its relative position in the human complex (Supplemental Figure S4B). This relative movement may also be driven by the substitution of K353 to H353, which in this new orientation interacts with Q498 (Fig. 1D). Importantly, previous data have shown that the K353H substitution substantially reduces SARS-CoV S protein binding to hACE2^45^.

The substitution of M82 to N82 in the ratACE2 and hamACE2 introduces an N-glycosylation site^45^. The effects of this glycosylation may be different in rats and hamsters. In fact, due to the altered orientation of the rat complex, the glycosylation may create steric hindrance with F486 and N487, in line with experimental data showing that M82N reduces SARS-CoV S protein affinity for ACE2^45^. In contrast, our model suggests limited impact of this glycosylation on the RBD-hamACE2 complex formation, in agreement with experimental data showing that hamsters can be infected by SARS-CoV-2 and transmit the virus to other hamsters^46^. However, experimental studies are needed to clarify the role of ACE2 glycosylation in these animal models.

Taken together, the differences in binding mode would suggest that mice and rat are unsuitable models for the study of COVID-19. Similarly, the presence of a salt bridge between K35 and E484 in the cavACE2-RBD complex (Supplemental Figure S3) would make guinea pig an unsuitable model for the study of inhibitory antibodies and small molecules targeting the ACE2 – SARS-CoV-2 S protein interaction.

### SARS-CoV S protein in complex with ACE2

**Figure 2:**
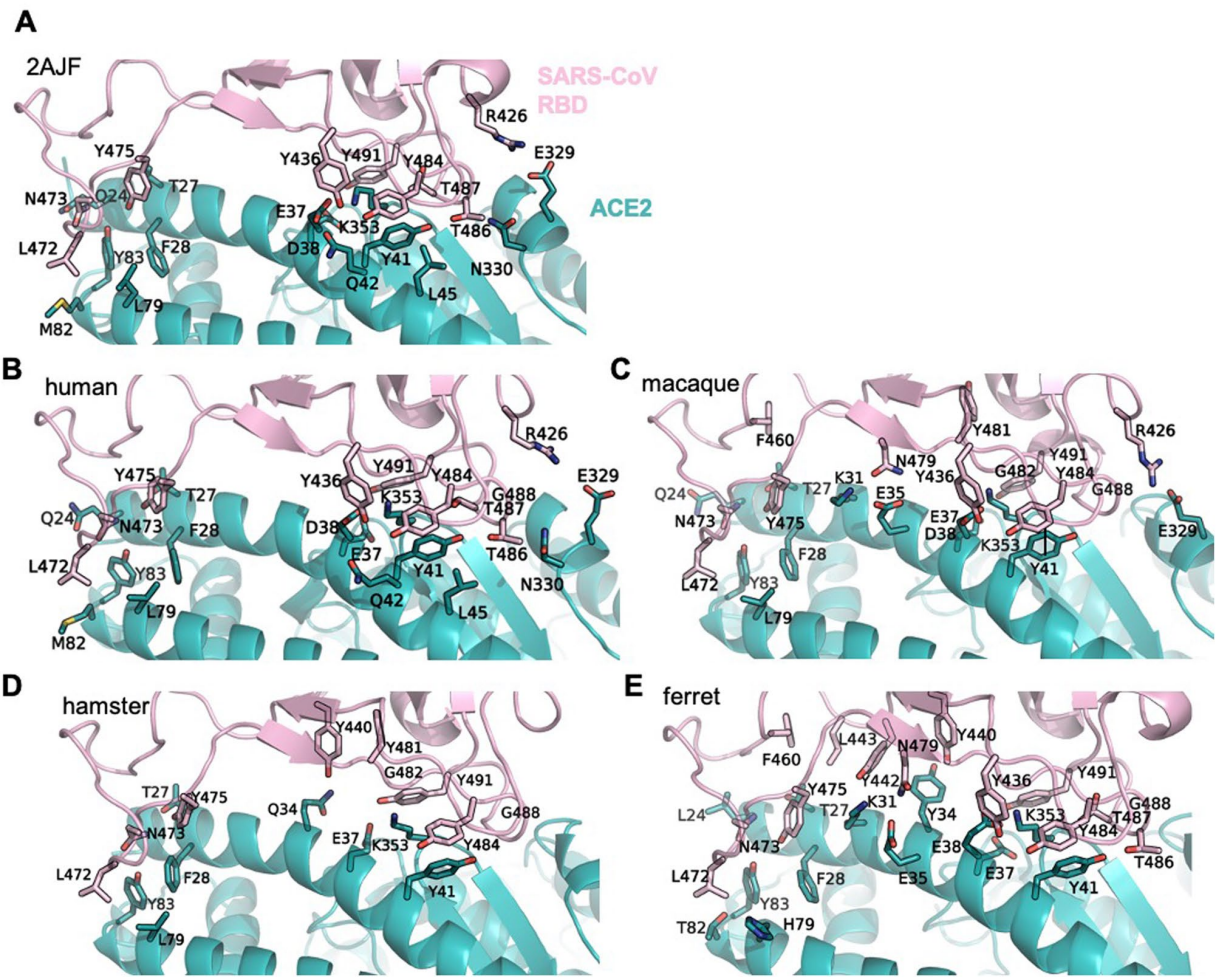
ACE2 PD – SARS-CoV S protein RBD interaction surface. Cartoon representation of the of the interaction surface of SARS-CoV RBD with ACE2 in the (**A**) X-RAY structure (PDB ID 2AJF), (**B**) human model, (**C**) macaque model, (**D**) hamster model and (**E**) ferret model. The residues involved in direct interactions (see Table 3) are shown as sticks, with the SARS-CoV RBD in pink and the ACE2 in teal. All structures are in the same orientation.

In order to further validate the approach adopted we carried out docking simulations between SARS-CoV RBD, for which more experimental data are available, and hACE2, cavACE2, dogACE2, catACE2, ratACE2, rabACE2, ferACE2, musACE2, hamACE and macACE2. Indeed, comparison of the SARS-CoV and the SARS-CoV-2 RBD in complex with ACE2 shows that the two co-crystal structures are comparable (RMSD 2AJF) and the binding interfaces are similar (Table 3 and Fig. 2A). In line with previously published data, we see that the SARS-CoV S has a smaller interaction surface and a lower number of interactions with ACE2 compared to the SARS-CoV-2 S protein (Figs. 1A, 2A)^47^.

**Table 3:** List of residues forming direct interactions in the ACE2 PD–SARS-CoV S protein RBD complexes.

	ACE2–SARS-CoV RBD										
	ACE2—RBD (PDB 2AJF)	hACE-RBD	cavACE2	dogACE2	catACE2	ratACE2	rabACE2	ferACE2	musACE2	hamACE2	macACE2
H-bond/polar interactions	Q24-N473, T27-Y475, E37-Y491, D38-Y436, Y41-T486, Y41-T487, Q42-Y436, Y83-N473, N330-T486, K353-G488	Q24-N473, T27-Y475, E37-Y491, D38-Y436, Y41-T486, Y41-T487, Q42-Y436, Y83-N473, N330-T486, K353-G488	Q24-N473, Q24-D463, E37-Y491, D38-Y436, Y41-T486, Y41-T487, Y83-N473, K353-T486, K353-T486	E38-Y436, Y41-T486, N330-T486	E37-Y491, E38-T484, Y83-N473, K355-G488	K24-N473, S27-Y475, K31-Y442, K31-N479, E35-N479, D38-Y436, Y41-T486, H353-G488	L24-N473, K31-Y442, K31-N479, E37-Y491, Y83-Y475, Y83-N473, K353-G488	L24-N473, K31-Y442, E35-N479, E37-Y491, E38-Y436, Y41-T486, Y83-N473, K353-G488	N24-N473, N34-Y440, E35-N479, E37-Y491, D38-Y436, H353-G488	T27-Y475, Q34-Y440, E37-Y491, Y83-N473, K353-Y481, K353-G482, K353-G488	Q24-N473, K31-N479, E35-N479, E37-Y491, D38-Y436, Y83-N473, K353-Y481, K353-G482, K353-G488
Salt bridge	E329-R426	E329-R426		E326-R426	E331-R426	K24-D463	E329-R426				E329-R426
Hydrophobic	F28-Y475, Y41-Y484, L45-Y484, L79-L472, M82-L472, K353-Y491	F28-Y475, Y41-Y484, L45-Y484, L79-L472, M82-L472, K353-Y491	F28-Y475, L34-Y440, L34-Y442, Y41-Y484, K353-Y491	L24-P462, L24-D463, F28-Y475, F28-N473, K31-Y475, Y34-Y440, Y34-Y442, Y41-Y484, L45-Y484, K353-Y491	L24-P462, L24-D463, F28-Y475, K31-Y475, H34-Y442, Y41-Y484, L79-L472	F28-Y475, Q34-Y440, Y41-Y484, H353-Y491, H353-Y484	L24-D463, L24-P462, T27-F460, T27-L443, F28-Y475, Y41-Y484, Y41-T487, L45-Y484, L79-L472, T82-L472, K353-Y491, K353-T487	F28-Y475, T27-F460, T27-L443, Y34-Y440, Y41-Y484, H79-L472, T82-L472, K353-Y491, K353-T487	N24-P462, T27, L443, T27-F460, F28-Y475, L45-Y484, Y41-Y484, T79-L472, H353-Y491, H353-T487	F28-Y475, Y41-Y484, L79-L472, K353-Y491	T27-F460, F28-Y475, Y41-Y484, L79-L472, K353-Y491
Can be infected		Yes	Yes^49^	/	Yes^23^	No/yes^28^	/	Yes^23^	Yes^28^	Yes^50^	Yes^63^
Present symptoms		Yes	Yes^49^	/	No/mild^23^	No^49^	/	Yes^23^	No/mild^28^	Yes^50^	Yes^63^

Residues forming contacts in the crystal structure (PDB ID: 2AJF) and in the HADDOCK docking models are listed by their position and by their single-letter identity, with the first residue belonging to ACE2 and the second to the S protein. Interactions were identified using PyMol and PDBePISA.

The mode of binding of SARS-CoV RBD to hACE2 has several differences compared to that of the other ACE2 proteins analysed. While the binding of RBD to hACE2 is driven by polar interactions, similarly to what we observed for SARS-CoV-2, in all other SARS-CoV RBD-ACE2 models there are fewer H-bonds and a concomitant increase in hydrophobic interactions. Importantly, the substitution H34 to Y/L34 introduces a steric interference, which results in a shift of ~ 3 Å of the CR1 loop of RBD^48^ bound to the dogACE2 and the cavACE2, compared to its relative position in the human complex (Supplementary Figure S6A, B). Similarly, the substitution of E329 with T/A/G/Q/K329 prevents the formation of a salt bridge with R426, and in the cavACE2 complex creates a charge repulsion (Supplementary Figure S6C). Overall, this would suggest a lower affinity of the SARS-CoV RBD for the cavACE2, dogACE2, catACE2, ratACE2, rabACE2, ferACE2, musACE2, hamACE2 in line with previously published data showing different susceptibility to infection of animal models^49^. Indeed, SARS-CoV infection in cats, ferrets, mice, guinea pigs, and rats is weaker and does not replicate the human disease in all its aspects^49^.

The interaction pattern of SARS-CoV with ferACE2 and hamACE2 is overall comparable to that of macACE and hACE2 (Fig. 2B–E). This is in line with experimental findings showing that hamsters support virus replication in the respiratory tract, have pronounced pathological findings in acute infection, but do not present symptoms^50^. Similarly, virus replication and pneumonitis were observed in ferrets infected with SARS-CoV. Ferrets do not develop fever, but presented pulmonary lesions similar, but milder, to macaques^23^. These milder symptoms in hamsters and ferrets could be linked to the missing E329-R426 bond present in human and macaque. In the same study, the authors reported even milder lesions in cats. This is in agreement with our data showing differences in the interaction pattern between the RBD and the hACE2 and catACE2 with a reduction in H-bonds^23^. Taken together our approach may provide a rationale for the observed experimental differences of the infection in human and animal models.

### TMPRSS2 and Furin are highly conserved across species

TMPRSS2 is a type II transmembrane serine protease (TTSPs), with an extracellular region composed of a low-density lipoprotein (LDL) receptor class A domain, a scavenger receptor cysteine-rich (SRCR) domain and a peptidase S1 domain containing the catalytic triad (Supplemental Figure S7). TMPRSS2, similar to other TTSPs, has high affinity towards substrates containing an Arg residue in the P1 position. Indeed, TMPRSS2 can recognise the SPRRAR/SVASQS and SKPSKR/SFIEDL sequences in the SARS-CoV-2 S glycoprotein and cleaves S1 from S2 between residues 685/686 and further cleaves S2 between residues 815/816, resulting in the formation of S2′^51^.

**Figure 3:**
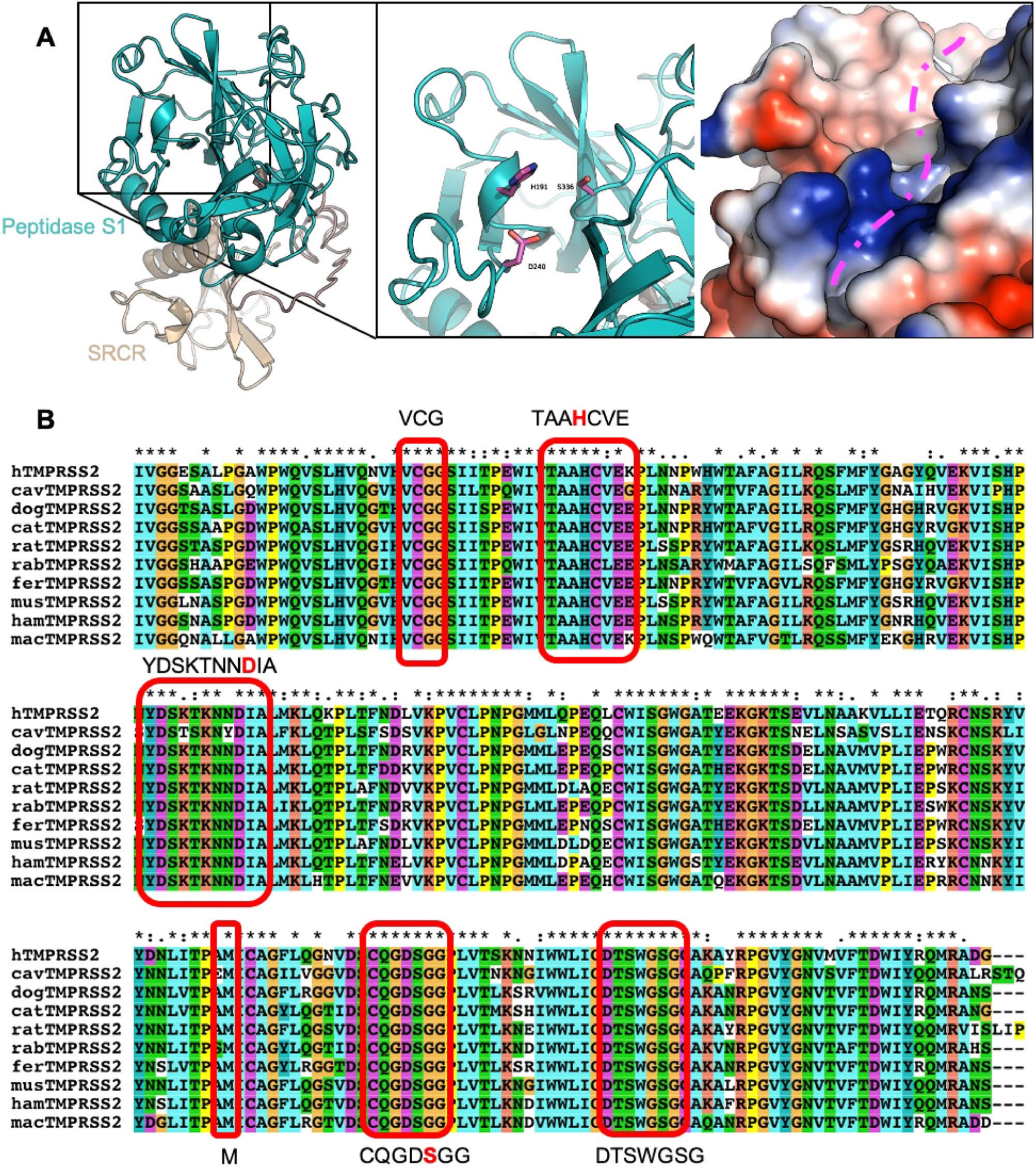
The TMPRSS2 active site is highly conserved among species. (**A**) Cartoon representation of hTMPRSS2, with the SRCR domain in beige and the Peptidase S1 domain in teal. In the close-up panels the catalytic triad (H296, D345 and S441) is shown in violet sticks and molecular surface colored by electrostatic potential (from − 44 kT/e (red) to 44 kT/e (blue)). A pink dotted line has been placed in the Peptidase S1 active pocket. (**B**) Multiple sequences alignment of hTMPRSS2, cavTMPRSS2, dogTMPRSS2, catTMPRSS2, ratTMPRSS2, rabTMPRSS2, ferTMPRSS2, musTMPRSS2. Peptidase S1 active pocket residues have been highlighted in red with the relative consensus sequence. Catalytic triad residues are shown in red in the consensus sequence.

In order to predict if TMPRSS2 from other animal species can cleave the SARS-CoV-2 S protein, the extracellular domain sequences of hTMPRSS2, cavTMPRSS2, dogTMPRSS2, catTMPRSS2, ratTMPRSS2, rabTMPRSS2, ferTMPRSS2 and musTMPRSS2 were aligned. The alignment revealed high conservation with a sequence identity between 75.11% and 83.97% (Supplemental Figure S2). We then generated a model for hTMPRSS2 (Fig. 3A and Figure S7) using I-TASSER, since classic homology modelling failed to identify a reliable template. The best I-TASSER output had a C-score of -0.52 with a TM-score of 0.65 ± 0.13 and an RMSD of 7.9 ± 4.4 Å. The model has the three conserved disulphide bonds on the peptidase S1 domain, characteristic feature of all TTSPs, between residues C281-C297, C410-C426 and C437-C465^52^. Disulphide bonds are also present between C113-C126, C120-C139, C133-C148, C172-C231 and C185-C241, which further validates the reliability of the models generated (Fig. 3A and S7).

The pocket containing the catalytic triad has a uniform negative charge, which favours electrostatic interactions with the Arg reach peptide of the S protein (Fig. 3A). Using this structural information, we identified the residues surrounding the catalytic triad that form a pocket on the head of TMPRSS2. Interestingly, this pocket is identical in all species studied and no substantial differences from the hTMPRSS2 were observed (Fig. 3B).

In addition to TMPRSS2, the protease Furin has also been shown to be involved in SARS-CoV-2 S protein cleavage at the S1/S2 site^12,13^. The S protein is initially cleaved by Furin and is then further processed by TMPRSS2 and so both proteases appear to be indispensable in viral entry^13^. We therefore also compared the well characterised substrate-binding residues of Furin^17^ across species. Similarly to TMPRSS2, Furin is highly conserved across species (94–98.9% similar to human, Figure S2). Further, the substrate-binding cleft is identical in all species studied (Supplementary Figure S8). Taken together these data suggest that TMPRSS2 and Furin of all species studied can cleave the SARS-CoV-2 S glycoprotein in a similar way to hTMPRSS2.

## Discussion

The rate at which new infectious diseases are discovered has dramatically increased in the last 20 years^53^. Most of the new viral infections are caused by viruses belonging to well-characterised virus families, like the novel coronavirus SARS-CoV-2^1^. SARS-CoV-2 was identified in December 2019 to be the viral agent causing COVID-19, which has now spread to over 100 countries^1^. As a result, there is a global priority to identify and develop effective vaccines and drugs for the treatment of the disease. However, this effort is in part being hampered by the lack of suitable animal models^27^. Ideal animal models should be infected in a similar way to humans, present comparable symptoms, present a correlation between disease severity and virus titer, have similar histopathologic changes, virus growth kinetics, and comparable levels of mortality^54^.

Coronaviruses are characterised by the presence of the S glycoprotein on the viral surface, which confer a unique crown-like morphology to the virion^55^. The S protein mediates both the attachment to the host cell and the fusion with the cell membranes^7^. As such, the S protein is the key element that determines cell tropism and the host range^56^. For cellular entry, the S protein binds to ACE2 and is primed by TMPRSS2, which promotes endocytic entry of the virus^15^. Importantly, ACE2 and TMPRSS2 appear to be widely expressed across mammals. Lung transcriptomic data is not available for all of the species investigated in this study. However, investigation of expression data for *Macaca mulatta*, *Rattus norvegicus, Mus musculus, Canis lupus familiaris and Oryctolagus cuniculus* demonstrated that ACE2 and TMPRSS2 are detectable in the lungs of these species (Expression Atlas^57^ and Bgee^58^). Further, these receptors were also found to be expressed in the lungs of multiple other animal species (e.g. *Bos taurus, Macaca mulatta, Ovis aries and Papio Anubis*).

An increasing body of evidence suggests that the tight binding of the S protein to ACE2 is the reason for the high person-to-person transmission rates and severity associated with this disease^11,12,45^. This is most evident when comparing the SARS 2002–2004 pandemic with the SARS-CoV-2 pandemic, with 8,098 cases versus over 16 million cases (26th July 2020) respectively. Our analysis demonstrated that the SARS-CoV and SARS-CoV-2 S proteins have substantial differences in their ACE2 binding motifs (50% identity), which results in an increased number of contacts between the SARS-CoV-2 S protein and ACE2. This correlates with the observed 10- to 20-fold higher affinity of the SARS-CoV-2 S protein for ACE2, compared to SARS-CoV S protein^11^.

It is clear that SARS-CoV-2 infection rates differ between species, and this is linked to the ability of the S protein to exploit ACE2 and proteases to enter the host cell^56^. Transfection of HELA cells with ACE2 from different species (human, Chinese horseshoe bat, civet, pig and mouse) demonstrated that the receptor from all species could be used for entry, except for mouse^2^. Further, Hoffman et al*.* used pseudotyped virus to demonstrate that SARS-CoV-2 can enter human and African green monkey cell lines and to a lesser extent dog and bat lines. In contrast, the virus was found to be unable to infect pig, cow, hamster and mouse lines^59^. The demonstration that SARS-CoV-2 is unable to utilise the mouse ACE2 for entry, correlates with our findings that demonstrates that key residues in the interaction surface differ between musACE2 and hACE2. The results presented here would suggest that SARS-CoV-2 can interact with hamACE2 and hence it is surprising that Hoffman et al*.* found that the pseudotyped virus was unable to enter hamster cells^59^. However, in agreement with our findings, Sia et al. have shown that golden hamster is a suitable in vivo model for the study of SARS-CoV-2, with the symptoms resembling a mild infection in humans^46^.

Our results show that the Spike protein recognises macaque, hamster, and ferret in a comparable way to human ACE2, in line with data showing high susceptibility of these animals to SARS-CoV-2 infection^27,46^. In contrast, we saw substantial differences in the binding mode of the SARS-CoV and SARS-CoV-2 S protein to guinea pigs, mice and rats ACE2. For example, guinea pigs can be infected with SARS-CoV^49^ (no data available for SARS-CoV-2) and we report that although SARS-CoV-2 S protein can bind guinea pig ACE2 (cavACE2), the mode of binding differs to hACE2. In fact, compared to the human protein, the RBD-cavACE2 complex has a reduction of four direct hydrophobic contacts, which reduces protein affinity^60^ and a concomitant extra charge reinforced hydrogen bond, which stabilises protein binding^25^. This results in a difference in binding kinetics between the RBD-cavACE2 and the RBD-hACE2. The different types of interaction stabilising the complexes are also likely to affect the ability of Protein–Protein Interaction (PPI) inhibitors to modulate, in a comparable way, the binding of RBD to hACE and cavACE. Therefore, while guinea pigs could be useful models for e.g. toxicity studies, they are unlikely to be suitable for the testing of inhibitory antibodies and small molecules targeting the ACE2 – S protein interaction surface.

Unlike ACE2, TMPRSS2 and Furin are highly conserved across the species analysed. Modelling of TMPRSS2 demonstrated that the catalytic triad (residues H296, D345 and S441 in hTMPRSS2) forms a negatively charged pocket, which favours electrostatic interactions with the positive charged peptides of the S protein. This region was found to be identical in all species analysed and it therefore appears that differences in infectivity across species is unlikely to be due to structural differences in TMPRSS2. Similarly to what we saw for TMPRSS2, Furin is highly conserved across species, with a sequence similarity of 95–98.9% compared to the hFurin. The identical Furin substrate-binding cleft also suggests a similar activity of the protease in all species analysed.

This study suggests that the macaque, hamster and ferret are currently the most suitable models for studies that aim to target the ACE2 – S protein interaction. Previous studies found SARS-CoV to be mildly infective in mice compared to humans and this was suggested to be as a result of differences between the structure of hACE2 and musACE2^44^. Importantly, transgenic expression of hACE2 in mice (K18-hACE2 Mice) resulted in SARS-CoV becoming a rapidly fatal disease in this model^61^. Similarly, Bao et al. demonstrated that hACE2 transgenic mice are susceptible to SARS-CoV-2, unlike wild-type mice, and the infection resembles aspects of the human disease^62^. The hACE2 mouse is therefore also a suitable model for SARS-CoV-2 studies. This also supports the findings of this study, which suggests that the high level of TMPRSS2 similarity between species does not appear to affect viral entry, but instead it is the species-specific differences in the structure of ACE2 that affects SARS-CoV and SARS-CoV-2 infectivity. Due to the similarities in TMPRSS2 and Furin between species, studies that aim to target these proteases have a range of suitable models available.

## Supplementary information

Supplementary information.
