# Supplementary material for: Structural and functional modelling of SARS-CoV-2 entry in animal models
Source: Sci Rep. 2020 Sep 28;10:15917. doi: 10.1038/s41598-020-72528-z (PMC7522990; doi:10.1038/s41598-020-72528-z)
Supplement: Supplementary file 1 — Supplementary information. [file 41598_2020_72528_MOESM1_ESM.pdf]

Structural and functional modelling of SARS-CoV-2 entry in animal models

Greg N. Brooke[1,2,3] & **Filippo Prischi**[1,2,3]

[1]School of Life Sciences, University of Essex, Colchester, CO4 3SQ, UK

[2]contributed equally

[3]corresponding authors: gbrooke@essex.ac.uk; fprischi@essex.ac.uk.

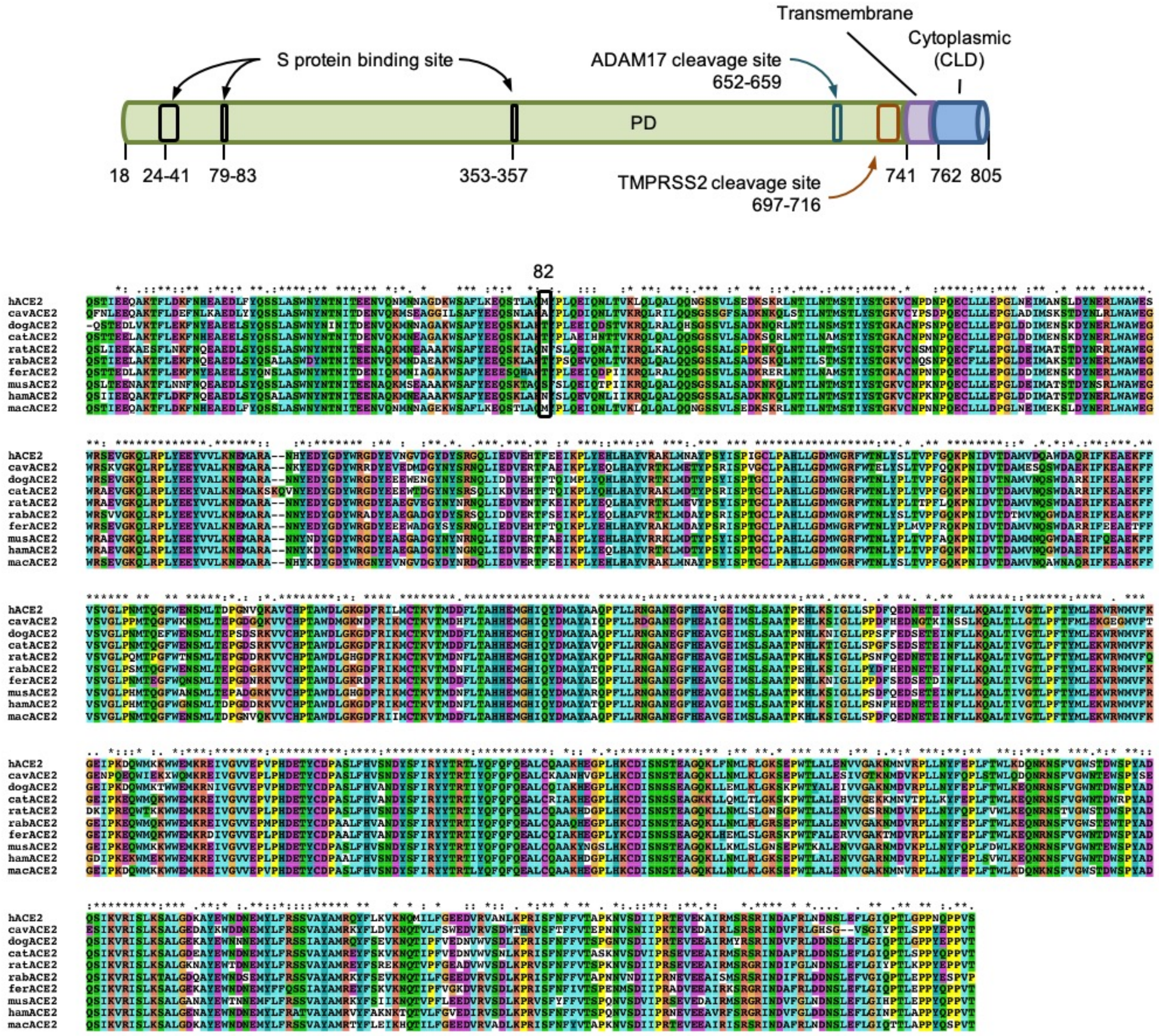

Figure S1 Schematic representation of hACE2. Peptidase Domain (PD) domain is shown in green, transmembrane region in purple and the cytoplasmic domain in blue. The S protein binding sites, ADAM17 and TMPRSS2 cleavage sites have been mapped on the model. Multiple sequences alignment was performed using Clustal Omega for hACE2, cavACE2, dogACE2, catACE2, ratACE2, rabACE2, ferACE2, musACE2.

**hACE2**	100									
**cavACE2**	78.5	100								
**dogACE2**	84.35	77.92	100							
**catACE2**	85.62	77.25	90.58	100						
**ratACE2**	83.4	78.5	82.69	82.99	100					
**rabACE2**	86.31	80.03	84.76	85.62	85.48	100				
**ferACE2**	82.85	76.28	89.47	89.07	81.88	84.09	100			
**musACE2**	82.99	79.33	83.24	83.26	90.04	85.34	83.26	100		
**hamACE2**	85.06	80.03	83.66	83.82	89.35	88.11	83.68	89.63	100	
**macACE2**	95.57	78.5	84.07	85.06	83.54	86.45	82.71	83.4	85.75	100
**hTMPRSS2**	100									
**cavTMPRSS2**	75.11	100								
**dogTMPRSS2**	83.54	76.79	100							
**catTMPRSS2**	83.97	75.11	91.14	100						
**ratTMPRSS2**	82.7	70.59	85.65	82.28	100					
**rabTMPRSS2**	78.48	75.95	81.86	83.12	83.54	100				
**ferTMPRSS2**	81.86	76.79	93.67	91.56	83.54	81.01	100			
**musTMPRSS2**	83.97	79.32	86.92	83.54	95.78	83.12	84.39	100		
**hamTMPRSS2**	83.54	79.32	85.23	82.7	91.98	83.12	83.97	92.83	100	
**macTMPRSS2**	83.97	73	84.81	81.43	82.7	78.9	81.43	83.12	84.39	100
**hFurin**	100									
**cavFurin**	94.84	100								
**dogFurin**	95.46	94.33	100							
**catFurin**	96.47	94.7	97.48	100						
**ratFurin**	93.95	93.19	93.06	93.94	100					
**rabFurin**	95.08	93.32	93.94	94.95	92.3	100				
**ferFurin**	95.84	94.72	97.6	97.35	93.32	94.58	100			
**musFurin**	93.95	92.56	92.55	93.43	96.97	92.17	93.06	100		
**hamFurin**	95.47	94.58	94.45	95.46	96.09	93.95	94.71	95.46	100	
**macFurin**	98.87	95.09	95.46	96.47	93.95	94.96	95.97	94.2	95.47	100

Figure S2 Sequences identity matrixes resulting from the ACE2 and TMPRSS2 multiple sequences alignments. Sequence alignment was performed using Clustal Omega using the HHalign algorithm and Gonnett as transition matrix.

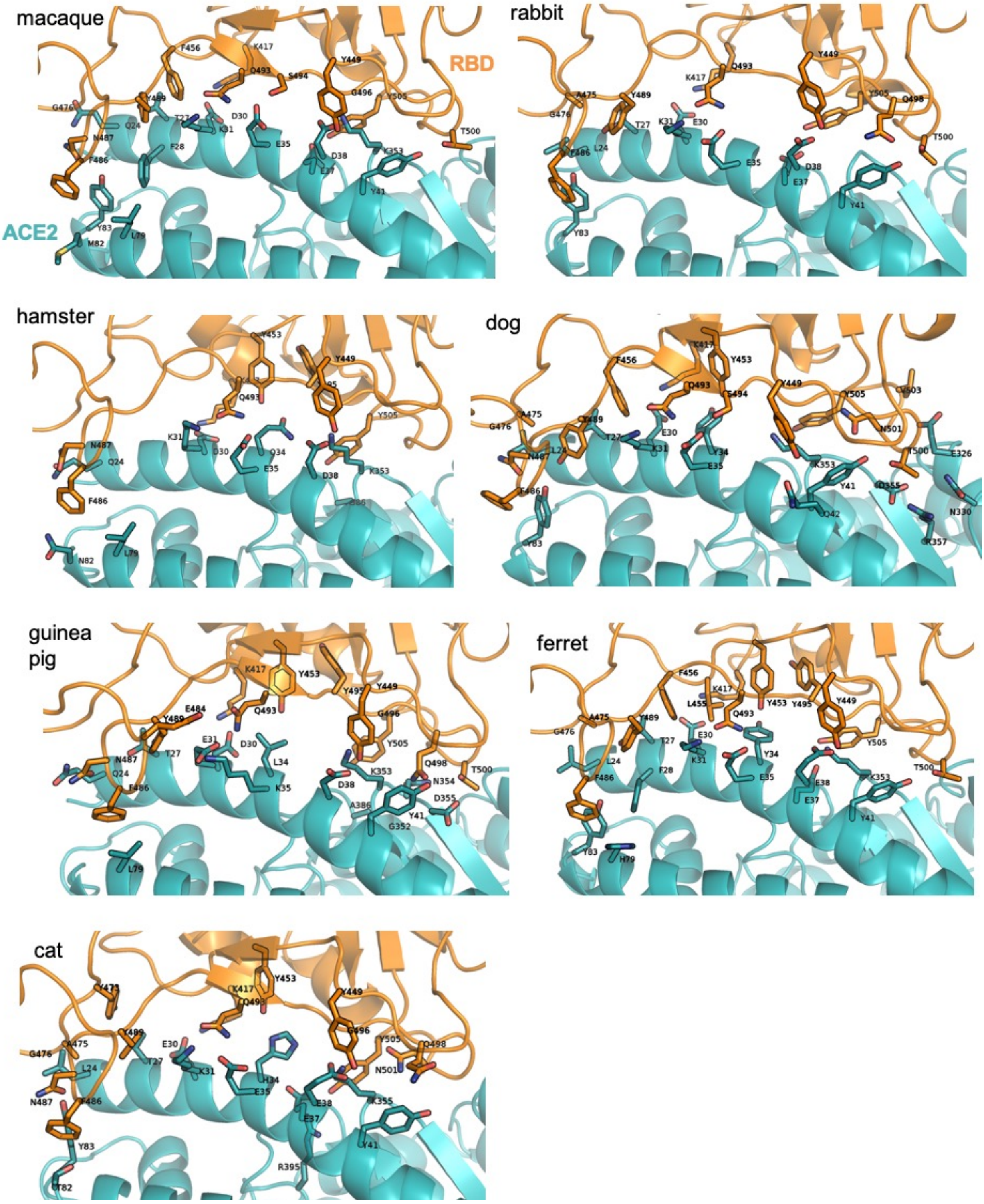

Figure S3 ACE2 PD – SARS-CoV-2 S protein RBD interaction surface. Cartoon representation of the of the interaction surface of SARS-CoV-2 RBD with the macACE2, rabACE2, hamACE2, dogACE2, cavACE2, ferACE2, catACE2. The residues involved in direct interactions (see Table 3) are shown as sticks, with the SARS-CoV RBD in orange and the ACE2 in teal. All structures are in the same orientation as in Figure 1.

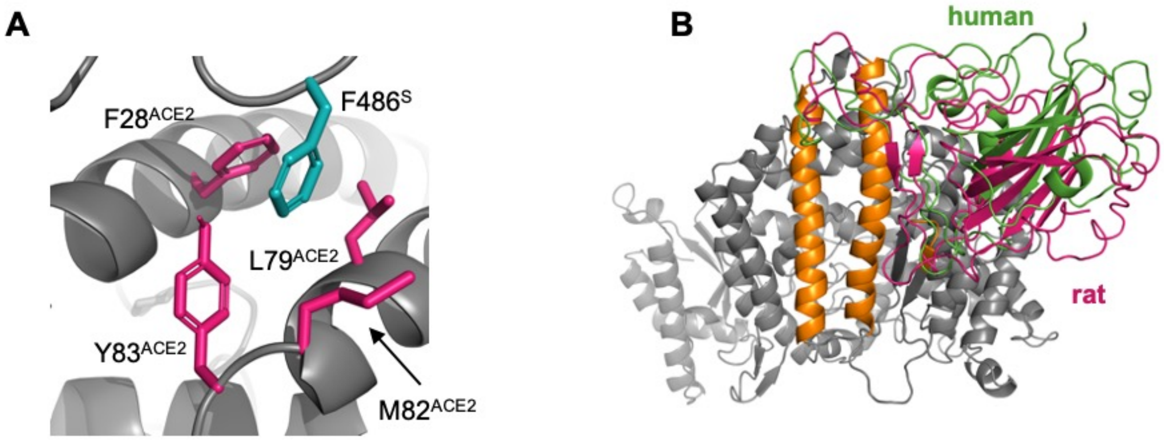

Figure S4 Cartoon representations of the SARS-CoV-2 RBD-ACE2 complex. (A) RBD F486 side chain (teal sticks) is inserted in a relatively hydrophobic patch formed by the hACE2 residues F28, L79, M82 and Y83, shown as hot pink sticks. **(B)** The structure of the hACE2-RBD complex is overlapped to the structure of ratACE2-RBD complex. Structures are shown in cartoon, with both ACE2 proteins in grey and α1, α2 helix and β3-β4 loop in orange. The RBD structure of the human complex is shown in green while the rat is in hot pink.

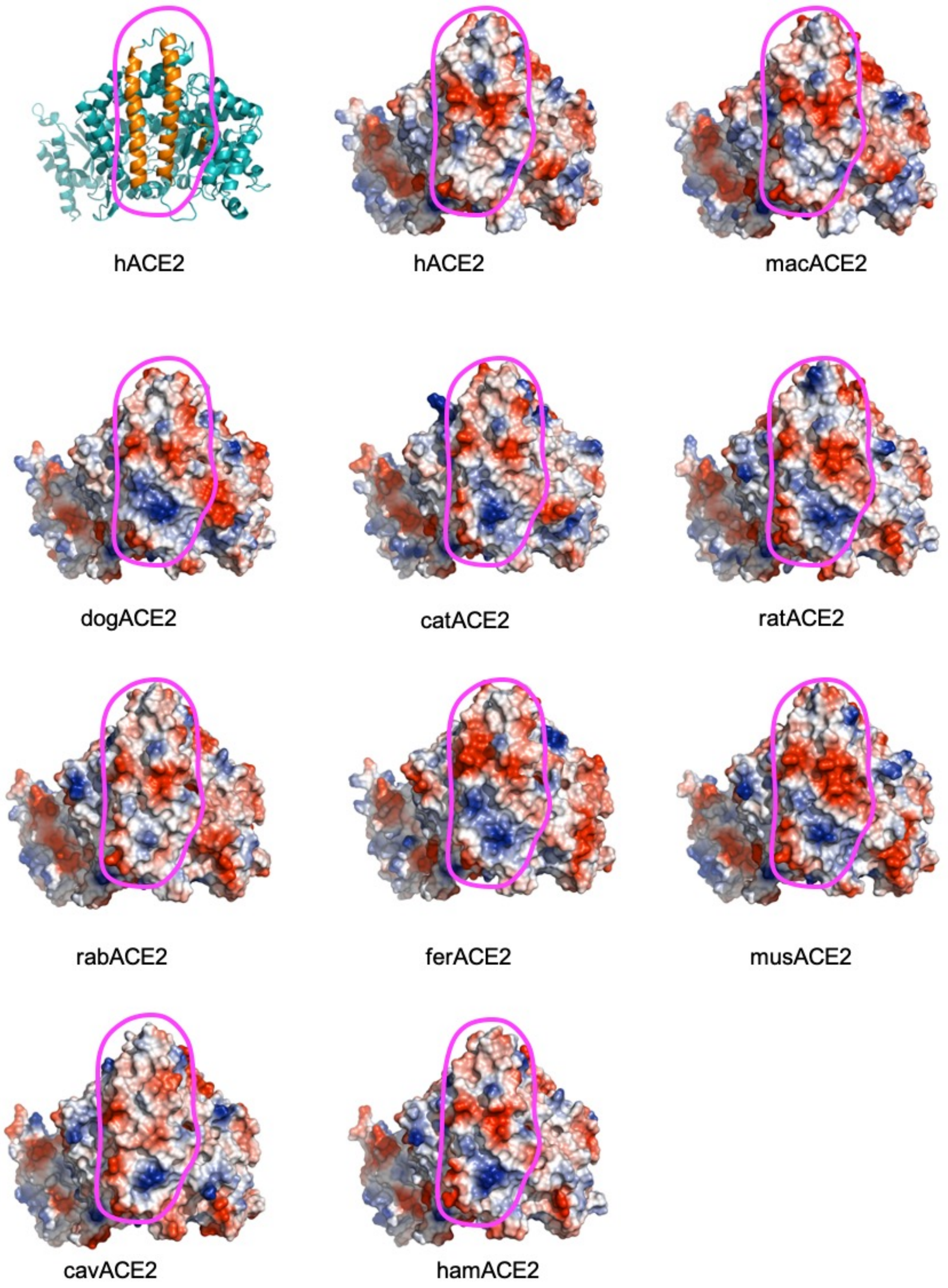

Figure S5 Structure of the ACE2 interaction surface across species. Electrostatic potential molecular surfaces representation of hACE-RBD, cavACE2, dogACE2, catACE2, ratACE2, rabACE2, ferACE2, musACE2, hamACE2 and macACE2 (from −68 kT/e (red) to 68 kT/e (blue)). The cartoon representation of hACE2 (in teal with α1, α2 helix and β3-β4 loop in orange) is used as reference. The RBD binding surface on ACE2 is highlighted in pink. All structures have been superimposed and are in the same orientation.

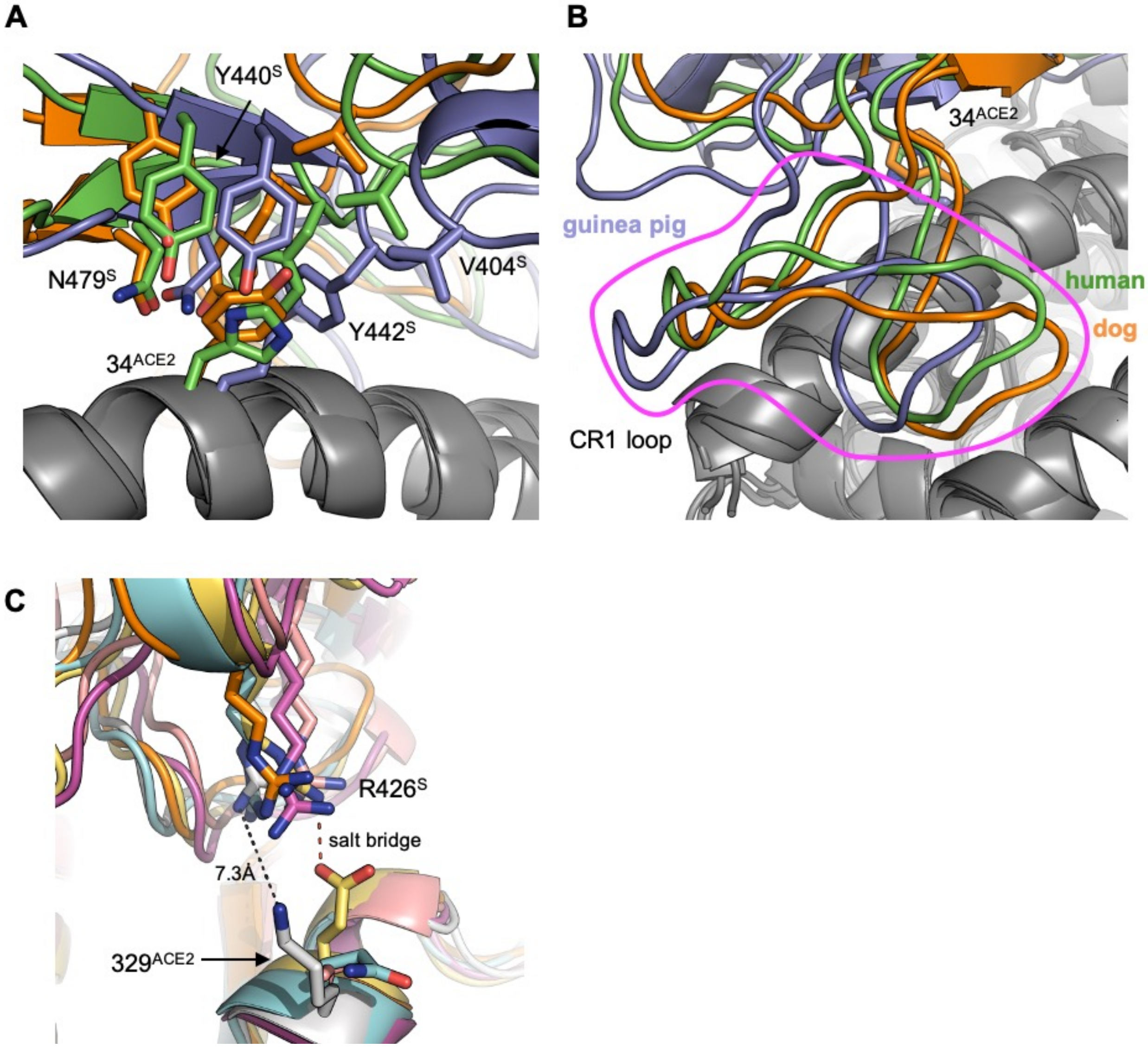

Figure S6 Cartoon representations of the SARS-CoV RBD-ACE2 complex. (A) The structure of the hACE2-RBD complex is overlapped with the cavACE2-RBD and dogACE2-RBD complexes. Structures are shown in cartoon, with S protein residues V404, Y440, Y442, N479 and ACE2 residue 34 in sticks. The human, guinea pig and dog complexes are colored in green, purple and orange respectively, and all ACE2 structures are colored in grey. **(B)** The CR1 loop of the RBD is highlighted in pink showing the relative shifts of the dog and guinea pig complexes respect the human structure. Same representations and colours as in panel (A). **(C)** The structure of the hACE2-RBD complex is overlapped to the structures of ferACE2-RBD, hamACE2-RBD, cavACE2-RBD, ratACE2-RBD and musACE2-RBD. Structures are shown in cartoon, with S protein residue R426 and ACE2 residue 329 in sticks. The E329-R426 salt bridge present in the human complex is shown as a red dotted line. The increased distance (7.3 Å) between the guinea pig K426 and the RBD E329, caused by electrostatic repulsion, is shown with a black dotted line. The human, ferret, hamster, guinea pig, rat, mouse complexes are colored in yellow, cyan, purple, white, pink and orange respectively.

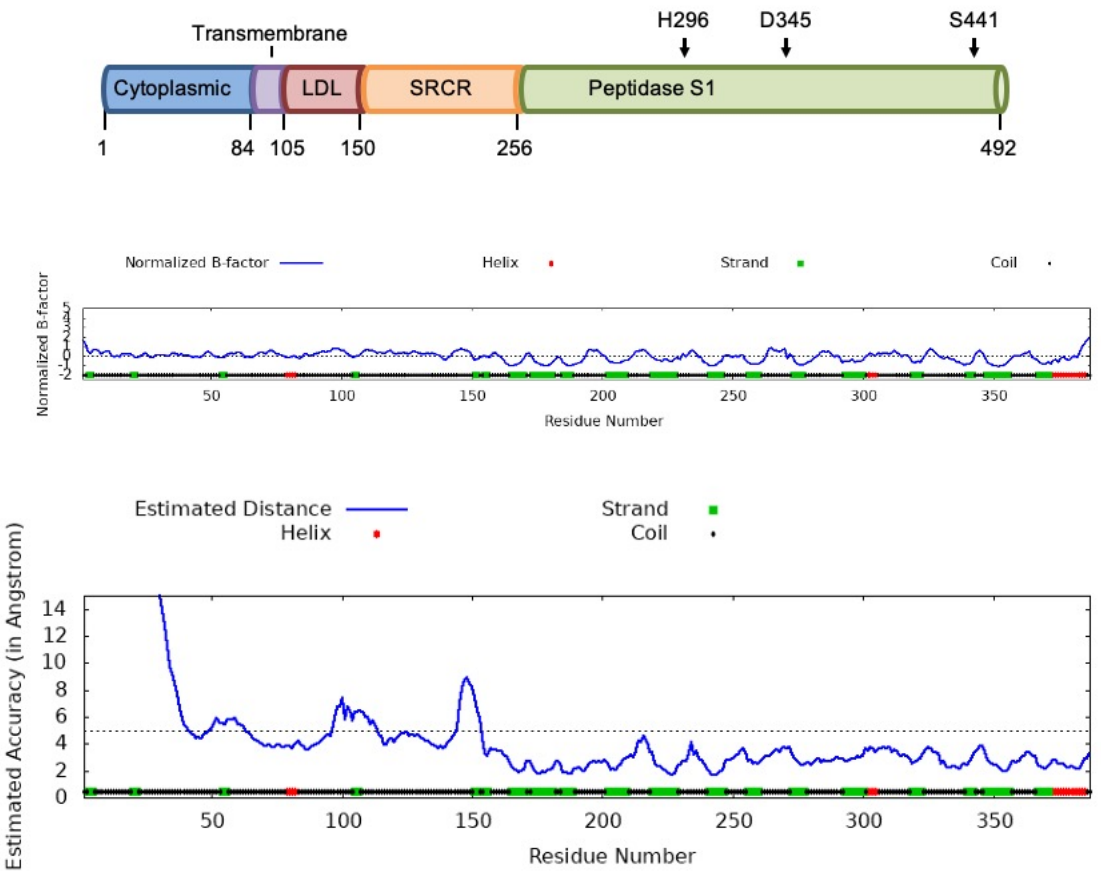

Figure S7 Schematic representation of hTMPRSS2. The cytoplasmic domain is shown in blue, the transmembrane helix is purple, the LDL domain in red, the SRCR domain in orange and the Peptidase S1 in green. The catalytic triad residues H296, D345 and S441 have been mapped on the model. Normalised β-factor and estimated accuracy plots of the I-TASSER hTMPRSS2 model. The estimated local accuracy shows that the LDL (1-150) has relatively higher modelling error while most of other regions are accurate with estimated distance to native smaller than 4 Å.

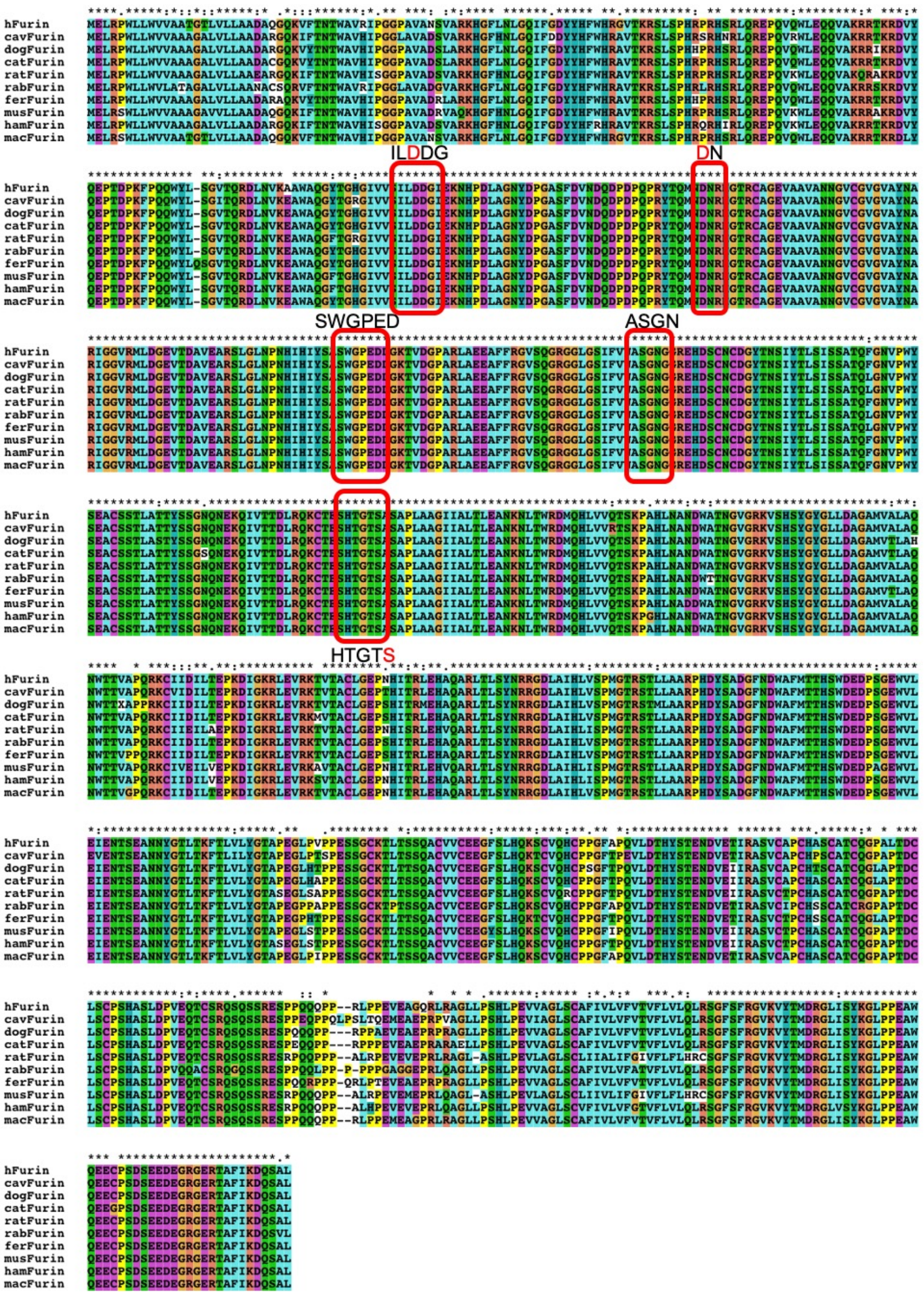
ILDDG
DN
SWGPED
ASGN
HTGTS
hFurin
cavFurin
dogFurin
catFurin
ratFurin
rabFurin
ferFurin
musFurin
hamFurin
macFurin

Figure S8 The Furin active site is highly conserved among species. Multiple sequences alignment of hFurin, cavFurin, dogFurin, catFurin, ratFurin, rabFurin, ferFurin, musFurin. Substrate-binding cleft residues have been highlighted in red with the relative consensus sequence. Catalytic triad residues are shown in red in the consensus sequence.
